# Numerical Study on Enhanced Line Focusing via Buried Metallic Nanowire Assisted Binary Plate

**DOI:** 10.3390/nano11020281

**Published:** 2021-01-22

**Authors:** Hyuntai Kim

**Affiliations:** Electrical and Electronic Convergence Department, Hongik University, Sejong 30016, Korea; hyuntai@hongik.ac.kr

**Keywords:** line focusing, binary zone plate, metallic nano-slit, nanowire, multi-focusing

## Abstract

Line focusing, which collects light into a line rather than a single point, has an advantage on variable fields such as machining and imaging. The 1-dimensional metallic zone plate is one of the candidates for line focusing, which is ultra-thin and simple to fabricate. Metallic nano-slits can replace the metal blocked region to increase the efficiency, however, the efficiency and stability are still low. Therefore, this paper proposes a structure with an additional dielectric layer to protect the metallic nano-slit layer—a buried metallic wire structure—and verify the idea based on numerical simulations. Two structures are proposed. In terms of stability, a flat surface structure is proposed and a corrugated surface structure with a consistent thickness with the nano-slit is proposed which has low fabrication difficulty. The optimization of the buried wire structure and performance after applying the buried wire structure to the dual-line focusing plate is calculated by numerical simulation. Finally, it was shown that the electric field intensity was 2.13 times greater.

## 1. Introduction

Focusing light has been studied and applied in various fields [1,2,3,4,5]. In particular, line focusing, which collects light on a single line rather than a single point, has an advantage in numerous fields such as laser machining, spectroscopy, imaging, and also solar concentration [6,7,8,9]. In addition, by controlling the focusing direction or focal point numbers, one could control the machining property or collect solar energy at multiple positions.

Another advantage of line focusing is the ease of lens fabrication—1-dimensional lens can generate line focus [9,10]. To greatly increase the ease of fabrication, one could think of a 1-dimensional binary metallic structure [11,12,13]. However, the metal binary structure has low efficiency, as it blocks the half portion of the phase [14,15]. To increase the efficiency, applying metallic nano-slit arrays enables us to use both portions of the phase in specific polarization conditions [15,16]. In previous research, the efficiency has been improved by assisting concentric sub-wavelength ring structure [16].

However, the 1-dimensional binary structure has no additional variable to control the efficiency, and it is also vulnerable to external damage due to the exposure of the metallic nanolayer. To compensate for such shortcomings, this paper proposes a buried metallic wire structure. An additional layer of protection is intended to add stability and improve efficiency. Fabricating a 1-dimensional metallic nano-slit on the surface and then depositing an additional protective layer over it would not only protect the metallic layer, but also give another order of freedom that can control the efficiency by adjusting the thickness of the additional layer.

In this paper, two structures are proposed. The first structure has buried nanowire arrays inside the dielectric layer. It is versatile and stable because of its flat surface, however, it has high difficulty on fabrication. The other structure is similar to the first, but the surface has the same distribution as the metallic layer—which means the additional dielectric layer is deposited after the metallic layer is engraved. The structure has an advantage on the fabrication process; however, the rough surface would show low durability or slight diffraction issue. After confirming and optimizing the characteristics of each structure, the efficiency is checked by making a dual-line focusing plate. Although there are various meta-surface lenses previously, it is notable that this structure is the simplest one-dimensional binary metal structure for fabrication, and it shows the advantage of not only efficiency compared to existing metal binary plates, but also the improvement of surface stability.

## 2. Materials and Methods

The metallic nano-slit structure exhibits properties similar to dielectric when the polarization and slit directions are perpendicular [15,17]. That is, even if a metal—relatively easy to process—is used, a binary lens with an efficiency comparable to that of a dielectric zone plate can be manufactured. However, when the metal is exposed to the surface, it is vulnerable to oxidation and external damage, and since the thickness of the metallic layer must be adjusted to match the phase condition, the resonance condition cannot be satisfied, so the transmittance is limited [18].

**Figure 1:**
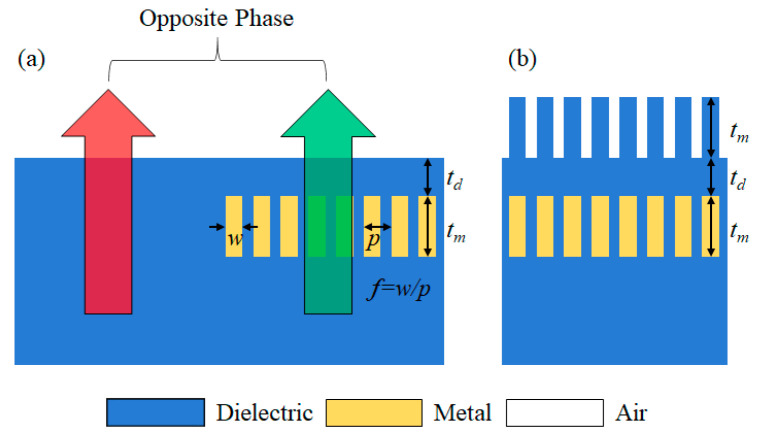
Schematic and operation principle of buried metallic nanowire structure. (**a**) Flat surface type, and (**b**) corrugated surface type.

As mentioned above, this paper proposes the following two structures. The schematic of the structure is shown in Figure 1. The phase of the part with the metal and the part without the metal are adjusted to be opposite, and the thickness of the protective layer is adjusted to optimize the transmittance. In the first type where the surface is flat which is shown in Figure 1a, the structure is relatively simple and endurable. However, the fabrication process would be complex—involving engraving the dielectric layer and filling it with metal, etching the upper side metal and then depositing the dielectric layer, or fabricating the second structure and polishing the surface. On the other hand, the second structure still has a corrugated surface, which causes diffraction and instability, but the fabrication difficulty is simple. After depositing and processing the metallic layer, additional dielectric layer deposition is required.

It is also available to use effective medium theory [19,20] for design optimization, but because there are several factors such as period and scattering, this paper optimized the structure with finite element method via COMSOL Multiphysics (COMSOL Inc., Stockholm, Sweden).

The wavelength is assumed to be 1064 nm, which is a typical ytterbium laser wavelength [21]. The incident sampling light was selected to be a Gaussian line beam with a beam waist of 4 μm, as the response of each small pixel is important. The material is assumed to be silicon dioxide (SiO_2_) [22] for dielectric and gold (Au) [23] for metal, which is proper to deposit via electron beam vapor deposition technique and to engrave pattern by focused ion beam milling, for example. The variables that can be controlled are the filling factor *f* (i.e., duty ratio), which is the ratio of metal within the period, the thickness of the metal, the thickness of the dielectric layer on the metal, and the period. In the case of the filling factor, the higher the metal portion, the lower the thickness of the metallic layer, resulting in lower stability and process difficulty, but it is less efficient because it blocks more parts or increases the imaginary refractive index of the layer from the point of view of effective medium theory. The larger the period, the lower the difficulty of the process, but if a few slits are allocated to each region in the future, the result is different from the expected characteristics, so it is assumed that the current processability is 100 nm. In the case of the filling factor, the smaller the value, the higher the transmittance. However, at a lower value, for example, less than 0.3, the metal remains very thin, with walls less than ~30 nm. In this paper, the filling factor was optimized between 0.5~0.7. This is a range that can be sufficiently manufactured at the current 10~40 nm process technology resolution, based on the assumed material. In the case of thickness, the calculation was performed within the maximum range of 1 μm. As the thickness increases, the stability of the array also deteriorates, and since resonance occurs within one wavelength order, optimization within a wavelength size is available and the structure can be kept thin. Note that both filling factor and period can be modified depending on conditions.

After optimizing the area, one can allocate the metallic region according to the phase of the desired lens or plate. However, since there is generally a degree of freedom in the phase, it is necessary to allocate the width of the open area to be maximum. If the intensity distribution of the incident light is known, one can optimize the total transmission amount after giving the intensity as a weight function.

## 3. Results

### 3.1. Wire Parameter Optimization

First, optimization of the geometry of the buried wire section that becomes the opposite phase was performed by tuning the parameters shown in Figure 1. The most important condition is that the phase difference should be opposite to that of the part without metal, Under the phase condition, the figure of merit is the transmittance. Both flat surface structure and corrugated surface structure were optimized.

Here a variable *Φ_D_* is defined. The phase difference *Φ_D_* is a figure of merit that represents the relationship between the phase of the metallic region and the dielectric region. Its equation can be expressed as follows:

(1) $$\Phi_{D}=∠E_{d}-∠E_{m}-\pi$$

where $∠E_{d}$ and $∠E_{m}$ represents the phase of the field after the dielectric region and buried wire region. The two regions exhibit opposite phases when *Φ_D_* is 0.

**Figure 2:**
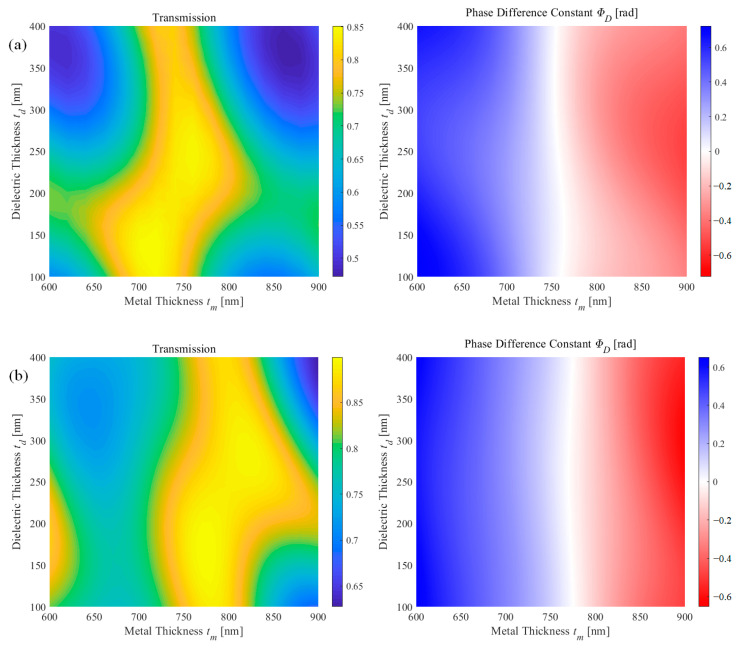
Transmission and phase difference constant in terms of metal thickness and dielectric for (**a**) flat surface structure and (**b**) corrugated surface structure.

Figure 2 shows the transmission and phase difference constant in terms of metal thickness and dielectric thickness on both flat surface and corrugated surface structure. Note that the period was fixed to be 100 nm, and another filling factor was calculated but the optimized case—0.6 for flat surface structure and 0.5 for corrugated structure—is shown. Further discussion about each parameter will be discussed in the next section. In the case of the flat surface, the structure was optimized at period of 100 nm, filling factor *f* of 0.6, metal thickness *t_m_* of 760 nm, dielectric thickness *t_d_* of 240 nm. The transmission was 0.845 and the phase difference was −0.0145 rad. The corrugated surface structure was optimized at period of 100 nm, filling factor *f* of 0.5, metal thickness *t_m_* of 770 nm, dielectric thickness *t_d_* of 240 nm. The transmission was 0.881 and the phase difference was less than 10^−4^ rad. As shown in Figure 2, the transmission tends to oscillate in terms of both variables. This is similar to the transmission in multiple layers. Assuming the periodic array layer as a single medium, the flat surface structure has four layers, and the corrugated surface structure shows five layers. Depending on the thickness of each layer, it is a result of the interference of the direct wave and twice-reflected wave. In the case of constructive interference, local maxima appear, and in the case of destructive interference, local minima appear.

The phase difference is determined by the presence or absence of the metallic layer and a corrugated layer. The thickness of the dielectric layer has no significant effect on the phase difference since both structures with and without metal propagates the same distance. In particular, when the metallic layer has resonance similar to a matching layer—the white region in Figure 2—the phase difference is independent of the thickness of the dielectric layer.

### 3.2. Lens Design

**Figure 3:**
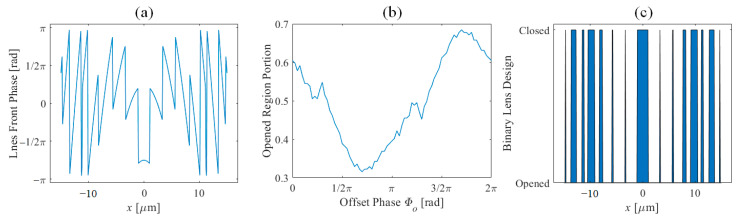
(**a**) Designed phase front of the dual-line focusing lens without offset phase. (**b**) The portion of opened area in terms of offset phase. (**c**) Optimized binary lens design.

This paper targeted the design of a dual off-axis line focusing 1D zone plate. The focal position is to be [−5, 20] μm and [5, 20] μm. The design was performed based on the virtual point method [24]. The lens front phase is shown in Figure 3a, while assuming the phase of the point is 0. As discussed, the phase of the lens is another controllable parameter, so an additional phase is assumed to the lens and the opening ratio of the total lens is calculated. Once more note that if the incident light intensity distribution is known, it is possible to optimize the transmission by giving the light intensity and metallic region transmission as a weight function. The opening portion in terms of offset phase *Φ_O_* is shown in Figure 3b and was optimized when the offset phase was 1.7 π. The binary lens structure considering the optimized offset phase is shown in Figure 3c.

Based on the calculated phase front, dielectric and buried wire region is assigned to a positive and negative phase, respectively. Three geometry was simulated. First, conventional metallic zone plate without any nanostructures as a comparative case. Two other structures are where the metallic is region replaced by the flat surface type and corrugated surface type structure, respectively. The incident light was illuminated from the bottom side, and the field distribution is assumed to be identical, such as a plane wave. The polarization of the electric field is the *x*-direction.

**Figure 4:**
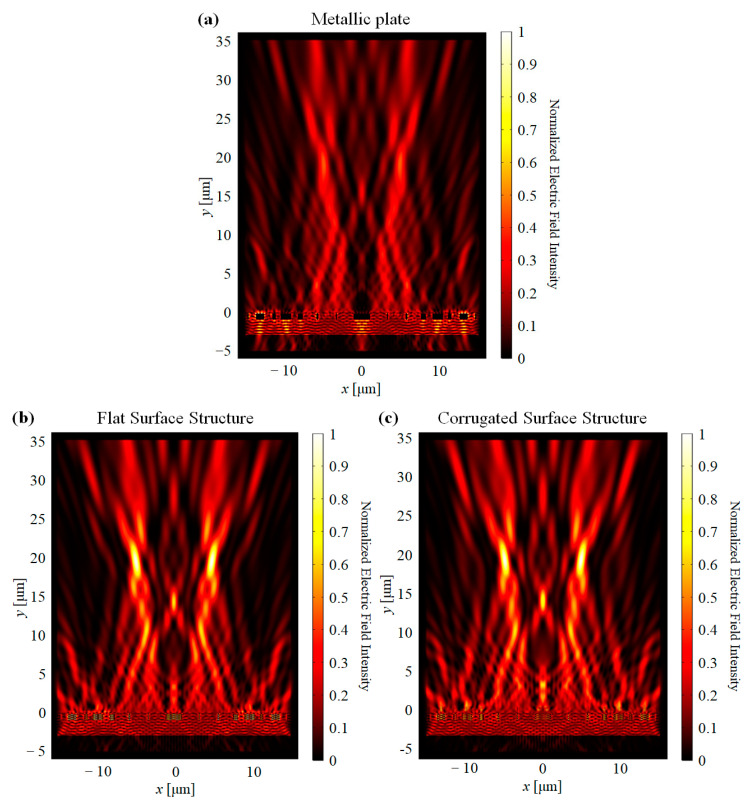
Normalized electric field intensity distribution of the dual-line focusing lens. (**a**) Metallic structure, (**b**) flat surface structure, and (**c**) corrugated surface structure.

As shown in Figure 4, the designed lens has focused light at the desired position. It is also clear that the buried wire structure enhances the feature of the focusing. The maximum field intensity was increased, 119.5% for flat surface structure and 119.3% for corrugated surface structure, which showed higher than twice of intensity compare to the metallic structure.

**Figure 5:**
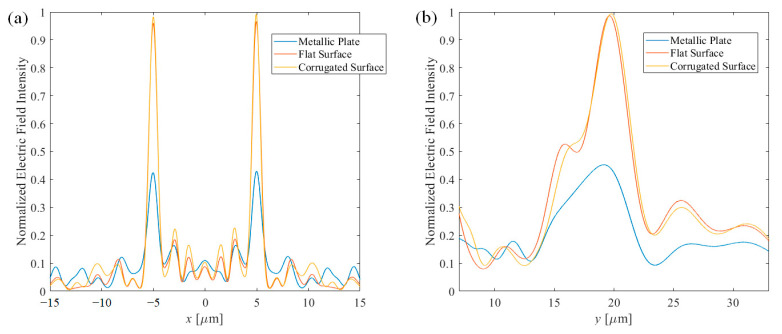
Normalized electric field intensity of metallic, flat surface, and corrugated structure at the (**a**) focal plane and (**b**) longitudinal axis.

The 1-dimensional plot of the electric field intensity was also plotted in Figure 5. Figure 5a shows the field intensity at the focal plane, i.e., *y* = 20 μm, while Figure 5b shows the longitudinal distribution, where *x* = −5 μm. It is shown once more that the efficiency was dramatically increased when the buried wire structure was applied. Then, the full-width half-max of the focused point was also calculated. The *x*-direction beam sizes—beam waist—are 1.22 μm, 0.98 μm and 0.95 μm for metallic structure, flat surface structure and corrugated structure, respectively. The *y*-direction beam sizes—depth of focus—are 7.11 μm, 6.22 μm and 5.63 μm for metallic structure, flat surface structure and corrugated structure, respectively. When the negative phase also becomes opened, more information of the original phase is given, therefore resulting in tighter focusing.

## 4. Discussion

**Figure 6:**
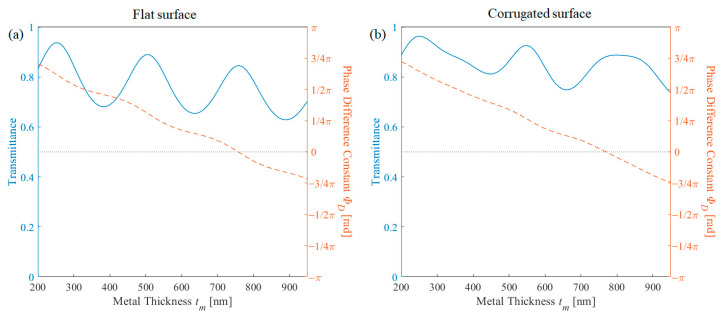
Transmission (blue solid line) and phase difference (red dashed line) in terms of metal thickness for (**a**) flat surface structure and (**b**) corrugated surface structure.

To analyze the effect of each parameter, the 1-dimensional transmission and phase difference plot is shown by modifying each factor. First, the transmission and phase difference was calculated by sweeping the metal thickness. Based on the effective medium theory [19], the periodic metallic layer could be regarded as an effective lossy dielectric medium. Therefore, when the thickness of the metallic layer increases, the phase change increases, the transmittance envelope decreases due to the small lossy factor, and it oscillates because of the resonance condition. Figure 6a shows the phenomena exactly, and Figure 6b shows non-periodic transmission behavior, as the corrugation layer thickness also varies.

**Figure 7:**
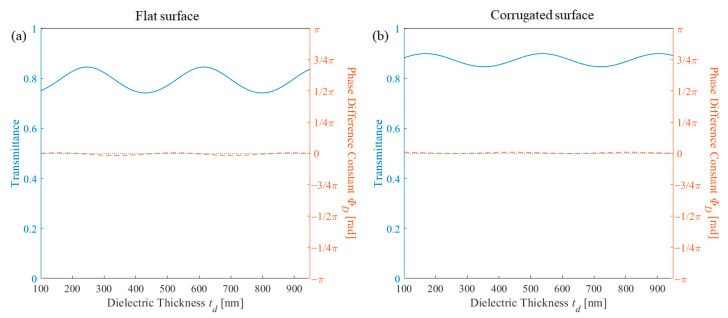
Transmission (blue solid line) and phase difference (red dashed line) in terms of dielectric thickness for (**a**) flat surface structure and (**b**) corrugated surface structure.

Then, numerical simulation was performed by varying the thickness of the dielectric layer and is depicted in Figure 7. As the dielectric layer is in common with the metal-free area, phase difference is independent of the dielectric layer thickness. The transmission oscillates, as expected. As mentioned above, the thickness of the dielectric layer adds a degree of freedom and could increase the transmittance. However, in this case, the metal region is in perfect phase matching. Note that on other parameter conditions shown in Figure 2, the phase difference also slightly varies dependent on the dielectric thickness when the phase difference constant becomes far from 0.

**Figure 8:**
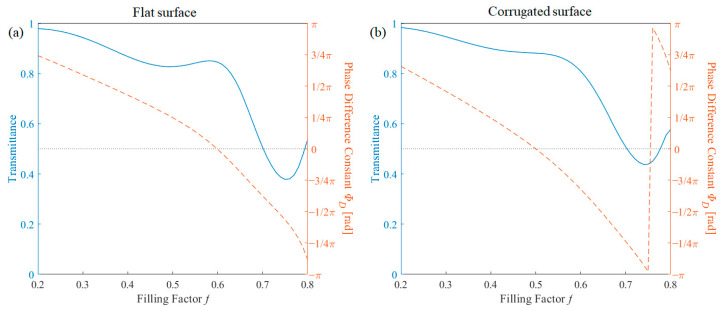
Transmission (blue solid line) and phase difference (red dashed line) in terms of filling factor thickness for (**a**) flat surface structure and (**b**) corrugated surface structure.

The effect of the filling factor was also calculated. Additionally based on effective medium theory, the higher the filling factor, both real and imaginary components of the wire region increase. Therefore, the transmission decreases when the filling factor increases, and also phase changes gradually because of the real part of the effective medium as shown in Figure 8. The transmission tends to have an oscillational component, which is caused by the resonance condition. As the transmission optimization was performed at a filling factor of 0.6 for the flat surface and 0.5 for the corrugated surface, the transmission shows local maxima at the designed value.

## 5. Conclusions

To conclude, two buried metallic wire structures were proposed to increase the efficiency of metallic zone plate lens. The flat surface buried wire structure has an advantage on stability, while the corrugated surface buried wire structure has an advantage on fabrication difficulty. Both structures were optimized and exhibited opposite phase compared to the region without wire and showed transmission of 0.845 for flat surface structure and 0.881 for corrugated surface structure. A dual-line focusing lens was designed and the enhancement from the buried wire was calculated as 119%. The buried metallic structure is expected to be applied on various 1-dimensional binary structure, and moreover to protect numerous metallic nanostructured thin-film optical components.

## Data Availability

Data is available within the article.
